# A comparative study of reaction times between type II diabetics and non-diabetics

**DOI:** 10.1186/1475-925X-4-12

**Published:** 2005-02-21

**Authors:** Samantha J Richerson, Charles J Robinson, Judy Shum

**Affiliations:** 1 Biomedical Engineering Department, Bucknell University, 1 Derr Dr. Lewisburg, Pa 17837 USA; 2 Center for Biomedical Engineering and Rehabilitation Science, Louisiana Tech University, 711 S. Vienna St, Ruston, LA 71270 USA; 3 Electrical Engineering Department, Bucknell University, 1 Derr Dr. Lewisubrg, Pa 17837 USA

## Abstract

**Background:**

Aging has been shown to slow reflexes and increase reaction time to varied stimuli. However, the effect of Type II diabetes on these same reaction times has not been reported. Diabetes affects peripheral nerves in the somatosensory and auditory system, slows psychomotor responses, and has cognitive effects on those individuals without proper metabolic control, all of which may affect reaction times. The additional slowing of reaction times may affect every-day tasks such as balance, increasing the probability of a slip or fall.

**Methods:**

Reaction times to a plantar touch, a pure tone auditory stimulus, and rightward whole-body lateral movement of 4 mm at 100 mm/s^2 ^on a platform upon which a subject stood, were measured in 37 adults over 50 yrs old. Thirteen (mean age = 60.6 ± 6.5 years) had a clinical diagnosis of type II diabetes and 24 (mean age = 59.4 ± 8.0 years) did not. Group averages were compared to averages obtained from nine healthy younger adult group (mean age = 22.7 ± 1.2 years).

**Results:**

Average reaction times for plantar touch were significantly longer in diabetic adults than the other two groups, while auditory reaction times were not significantly different among groups. Whole body reaction times were significantly different among all three groups with diabetic adults having the longest reaction times, followed by age-matched adults, and then younger adults.

**Conclusion:**

Whole body reaction time has been shown to be a sensitive indicator of differences between young adults, healthy mature adults, and mature diabetic adults. Additionally, the increased reaction time seen in this modality for subjects with diabetes may be one cause of increased slips and falls in this group.

## Background

Aging slows reflexes and increases the time to react to a number of external stimuli of different modalities [1-4]. What has escaped extensive examination has been the effect of Type II diabetes on these same reaction times and the comparison of modalities across the various sensory inputs. Only two studies have tested older individuals with diabetes. These have demonstrated increased reaction times to visual and auditory stimuli [5,6]. Mohan, et al. [5] found a 30 ms difference in auditory reaction times between those with diabetes (approximately 210 ms) and a control group (180 ms). Dobrzanski, et al. [6] found a doubling of visual reaction time in diabetics (473 ms) versus that measured in healthy individuals (216 ms). In addition to the measured effects in these two studies, diabetes has also been shown to affect peripheral nerves in the somatosensory [7] and auditory system [8], slows psychomotor responses [9], and has cognitive effects on those individuals without proper metabolic control [10-13], all of which may affect reaction times.

One of the largest implications that an increased reaction time may have is in the area of slips and falls. Falls are incurred by one third of the elderly population and are a common source of morbidity and mortality[14]. Evidence that older subjects have an increased incidence of slips and falls when compared to healthy young adults have been attributed to increase in sway as seen by center-of-pressure or -of-gravity (COP, COG), or head and hip variability [15,16]. Although no age related changes have been found in the rms distance of the anterior-posterior (AP) COP, changes in both mean velocity and range of AP and medial-lateral (ML) COP have been seen, with stronger changes in the former [17-20]. Diabetics have been shown to have a higher incidence of postural instability [21-26], and reduced peripheral sensations thus leading to an even higher incidence of falls resulting from slips than their healthy elder counterparts. These changes in balance metrics due to both normal aging and diabetes have been well measured, but never accurately explained. It is our contention that the postural instability may be due to slower input of information to the central nervous system, which does not allow the nervous system to react to stimuli as quickly, producing a higher incidence of slips and falls.

The aim of this study was to measure and compare reaction times to plantar touch, auditory tone, and whole body lateral movement in subjects over 50 years old with and without diabetes, as well as a group of healthy younger adults under 25 years of age. Subjects with diabetes were expected to have reaction times longer than those of the age-matched controls, while the aged controls were in turn expected to have reaction times greater than those seen in the younger adult group. The implications of the changes in reaction time will be discussed with respect to the central and peripheral nervous system.

## Methods

### Subjects

Subjects included 37 mature adults over 50 yrs old. Thirteen had a clinical diagnosis of type II diabetes made by their primary physician (group PN, mean = 60.6 ± 6.5 yrs, 7 Female/ 6 Male) and 23 did not (group NI, mean = 59.4 ± 8.0 yrs, 11 Female / 12 Make). The majority of the subjects were recruited from within the Veterans Administration (VA) population at the Overton Brooks VA Medical Center. Reaction times from these groups were compared to a younger adult group (age <25, N = 9, mean = 22.9 yrs, 4 Female/ 5 Male) that were recruited through advertising at Louisiana Tech University, and tested at the VA Medical Center. The recruiting, screening, testing and informed consent procedures were reviewed and approved by the local VA Institutional Review Board.

### Screening

Subjects recruited for this study were relatively healthy individuals with no current or past history of severe heart, circulation, or breathing problems; chronic lower back pain or spasms; deformities of the spine, bones or joints (including advanced arthritis); cerebral stroke, spinal cord injuries or other damage to the nervous system; non-healing skin ulcers; advanced diabetes; current drug or alcohol dependence; or repeated falls. Individuals taking any prescription medicine to prevent dizziness were also excluded.

Diabetic individuals targeted for this study were those with very early and mild Type II Diabetes. The subject's primary care physician undertook the diagnosis of diabetes. Targeted recruits had all been diagnosed within the last 10 years. All subjects with diabetes were using either diet or oral medication to manage blood sugar levels.

Visual, vestibular, muscoskeletal, and cognitive screening was also done to ensure that no undiagnosed problem existed that would prevent subjects from completing the study.

Plantar sensory tactile threshold were measured on each sole for all subjects using graded Semmes-Weinstein Monofilaments, which, upon bending, exerted a known force that depends on the filament diameter. Tactile force perception thresholds on the glabrous skin of the feet were determined for the right and left feet using these monofilaments according to standard clinical testing protocol. Stimuli were presented randomly three times at a given location, and, if two of the three presentations were detected, a threshold force was considered determined. Although force is a ratio metric (a measure in which an absolute zero is present and meaningful fractions or ratios can be constructed), the measurement of force by this method is still an ordinal (or rank ordered) type of data. Therefore, non-parametric statistics were used to compare tactile thresholds among test locations on the foot, and between right and left feet, as well as to compare among groups.

A certified audiologist at the Overton Brooks VA Medical Center carried out air conduction auditory threshold testing on all mature subjects (but none of the younger adults due to their health). Both mature adult groups underwent testing at 1, 2, 4, and 8 kHz in both ears. Average threshold level was recorded in decibels. Using a One Way ANOVA on Ranks, the threshold in each ear was compared to determine any differences in threshold.

In addition to this screening, all of the mature subjects underwent clinical surface nerve conduction studies of the lower extremity which were performed at the Neurology Service of the Overton Brooks VA Medical Center by a technician under the supervision of a neurologist. Motor (peroneal and tibial nerve) and sensory nerves (sural nerve) were tested bilaterally. F- and M- latency tests that test the entire lower motor loop (sensory nerve -> vertebrae -> motor nerve) were initially performed to ascertain any problems in the Sherrington's final common pathway [27]. However, the first two subjects expressed severe discomfort in undergoing that part of study. Hence the F- and M- latency tests were optional to subsequent subjects. These tests found peripheral neuropathies in all diabetics and none of the remaining mature subjects, who were thus classified as neurologically intact.

### Reaction Time Protocol

Reaction time was defined as the time between a stimulus onset and a signaled response of the subject. Three different stimuli were presented – touch, tone and platform movement.

A manually held, miniature single axis force sensor (Sensotec, Inc) with a 2 mm diameter tip was used by the authors to apply a tactile stimulus to the plantar surface of the big toe of each foot. Since the unloaded sensor force could vary over time or with a change in the position of the sensor, the single axis force sensor was calibrated to a zero state prior to each reaction time test series. A force change of more than 0.01 N was determined to be the trigger for an event. Instructions given to the subjects were to "press the button as soon as you feel the sensory touch the bottom of your foot." Subjects signaled detection of the stimulus via hand held bell button press. The latency between the onset of the rise in applied force measured by the force sensor and the resultant bell-press signal was taken to be the reaction time. Reaction times were measured five times and all trials were averaged. For auditory latencies, a bell tone was presented bilaterally via earphones and a subject signaled by pressing the force sensor with the thumb when he heard the signal. A change of approximately 10 times the sensor's resolution (0.01 N) or greater was determined to be the trigger for the detection of the event. Again, reaction time was averaged over all 5 trials.

Finally, a reaction time to a rightward lateral platform movement of 4 mm at 100 mm/s^2 ^was measured, while a subject stood barefoot on the platform with feet in normal stance. The SLIP-FALLS platform [28] was used to induce these movements because it produced smooth, precisely controlled, low vibration translations. Subjects were blindfolded and instructions were presented over a white noise background to the subjects though headphones. Subjects signaled detection of the movement though the use of a hand held push-button remote. Reaction times were averaged over ten trials.

## Results

All data sets analyzed failed a normaility test, prompting the authors to use non-parametric tests. In cases where two groups were compared, a Mann-Whitney Rank Sum Test was used. In cases where three or more groups were compared, a Kurskal-Wallis One Way ANOVA was used. The non-normality of the data also precluded the use of two-way ANOVAs. For all tests, the level of significance used was p < 0.05.

### Thresholds

#### Tactile Thresholds from Semmes- Weinstein Monofilament Tests

Table 1 gives the average force necessary for detection of each group tested at each location on the foot sole. None of the diabetics in this study had significant plantar sensory loss. No significant differences were found in thresholds between right and left legs for the metatarsal and toe in any group. Data from the right and left legs were then pooled. A non-paramedic (Kruskal-Wallis) one way ANOVA was used to determine difference in tactile threshold among groups. For both plantar locations, young adults had significantly lower thresholds (median = 0.07 N) than the other groups. The diabetic (median = 3.610 N for metatarsal and median = 3.220 N for toe) and healthy adult groups (median = 3.610 N for both plantar locations) did not differ significantly.

**Table 1:** Plantar, Auditory, Nerve Conduction and Reaction Time Metrics. Average reaction times to a plantar touch, a pure tone auditory stimulus, and rightward lateral movement of 4 mm at 100 mm/s^2 ^in diabetic (Peripheral Neuropathy), non-diabetic (Neurologically Intact), and young adults. Metrics given in either average ± standard deviation format or median [25% quartile, 75% quartile] format. All metrics for Semmes-Weinstein Monofilament Threshold, Air Conduction Threshold, and Nerve Conduction Studies have been averaged over both the right and left sides of the body. NCV = Nerve Conduction Velocity

**Group**	**Peripheral Neuropathy (n = 13)**	**Neurologically Intact (n = 24)**	**Young Adults (n = 9)**
**Age (yrs)**	60.6 ± 6.5	59.4 ± 8.0	22.7 ± 1.2

**Tactile Semmes-Weinstein Monofilament Thresholds (N)**			
Base Metatarsal	3.610 [2.473, 4.000]	3.610 [2.440, 3.610]	0.07 [0.0200, 0.160] †
Big Toe	3.220 [2.220, 4.080]	3.610 [2.440, 3.840]	0.07 [0.0200, 0.160] †

**Air Conduction Thresholds (dB)**			
1 K Hz	20.0 [15.0, 22.5]	15.0 [10.0, 20.0] †	
2 K Hz	20.0 [15.0, 30.0]	15.0 [10.0, 22.5] ‡‡	
4 K Hz	30.0 [17.5, 55.0]	25.0 [20.0, 35.0] ‡	
8 K Hz	35.0 [22.5, 67.5] ‡	35.0 [20.0, 57.5] ‡	

**Peak Accel Thresholds (mm/s^2^) to Platform Lateral Moves**			
1 mm	122.775 ± 68.964‡	108.417 ± 59.050 ‡	60.778 ± 51.832‡†
2 mm	77.407 ± 55.982 ‡†	42.664 ± 37.754 ‡†	10.386 ± 3.091†
4 mm	37.275 ± 30.341	18.572 ± 19.143	13.458 ± 8.343
8 mm	20.297 ± 18.680	14.077 ± 8.122	14.766 ± 8.012
16 mm	18.613 ± 9.790	11.258 ± 7.723	13.590 ± 9.083

**Nerve conduction studies**			
Sensory NCV (m/s) – Sural	41.0 [37.0, 45.0] †	45.0 [42.0, 47.0] †	
Motor NCV(m/s) Peroneal	42.0 [39.0, 46.0] †	48.0 [46.0, 49.0] †	
Motor NCV(m/s) – Tibial	40.5 [36.0, 45.0] †	45.0 [42.25, 48.75] †	
M-wave Latency (ms) – Peroneal	4.7 [4.15, 5.45]	4.6 [4.075, 5.3]	
M-wave Latency (ms) – Tibial	5.35 [4.4, 7.0] *	4.8 [4.2, 5.6] *	
F-wave Latency (ms) – Peroneal	52.7 [47.625, 60.950] †	49.7 [46.6, 52.325] †	
F-wave Latency (ms) – Tibial	58.5 [52.6, 64.7] †	52.5 [50.450, 55.275]†	

**Reaction Times (ms)**			
Touch (Big Toe)	353.1 ± 113.6	331.5 ± 140.5	216.0 ± 64.3
Tone (1 kHz)	282.6 ± 65.2	276.9 ± 105.5	218.6 ± 64.3
4 mm Lateral Platform Movement @ 100 mm/s^2^	777.8 ± 243.0‡†	623.9 ± 191.4‡†	431.5 ± 59.1‡†

†Indicates significant group difference

‡Indicates significant threshold difference

*Indicates a trend to significant threshold difference (0.05 < p < 0.07)

#### Audiology Thresholds

Because no significant difference (p = 0.481) was found between the right and left ears for all groups, the results of the air conduction testing of both ears were pooled and compared between groups. Averages for each group at each frequency (1, 2, 4, and 8 kHz) can be seen in Table 1. Significant differences were found both between groups and among frequencies. Diabetics had significantly higher thresholds at 8 kHz (median = 35.0 dB) and the healthy adult group had significantly higher thresholds at 4 and 8 kHz (median = 25.0 dB and 35.0 dB respectively). Additionally, there was no significant difference between the diabetics and non-diabetics at 4 and 8 kHz, but there was a significant difference at 1 kHz, and trend toward significance at 2 kHz (p = 0.055).

#### Thresholds to Rightward Lateral Platform Movements

Determining the minimum acceleration threshold required to detect motion requires special psychophysical test procedures. These procedures, results, and conclusions arising from such testing are complex enough to require treatment in entirely separate papers [29,30]. In summary, peak acceleration values required at threshold are a function of the displacement traveled and the group studied. These values are listed in Table 1. These results show that the lateral perturbation test used in this study (4 mm at 100 mm/s^2^) is well above the detection threshold of any of the three groups at 4 mm (~ 40.0 mm/s^2 ^for diabetics, and ~ 14.0 mm/s^2 ^for healthy mature and younger adults). Hence we have termed this stimulus a Superthreshold stimulus.

### Lower Limb Nerve Conduction Testing

Data was again pooled for both legs because all nerve conduction studies showed no differences between the two legs (values for each group can be seen in Table 1). Significantly slower conduction velocities (p < 0.05) were found for the sural, tibial, and peroneal nerves of the diabetic group. No significant differences was seen in the M latency of the peroneal nerve (p = 0.492) between groups, but a trend towards significance was seen in the tibial nerve (p = 0.07). The F latencies of both the peroneal and tibial nerves of the diabetic group were significantly higher than the healthy adults.

Although there was a significant slowing found within the diabetic group, the deficit present was not judged as severe by a trained neurologist. According to standards set forth by the VA Medical Center, normal motor nerve conduction studies have velocities greater than 44.0 m/s for the peroneal nerve and greater than 41.0 m/s for the tibial nerve. The median conduction velocities for adults with diabetes are 42.0 m/s and 40.5 m/s for the peroneal and tibial nerve respectively. These values are not within the normal range for nerve conduction studies (NCS), yet they do not represent severe slowing. This indicates that those in this study do not have advanced motor deficits, and that the extent of the peripheral neuropathy is significant, yet not severe and debilitating. Also, as expected, sensory nerve conduction velocities were slower than motor nerve conduction velocities, which validate the data.

### Reaction Time Measurement

**Figure 1:**
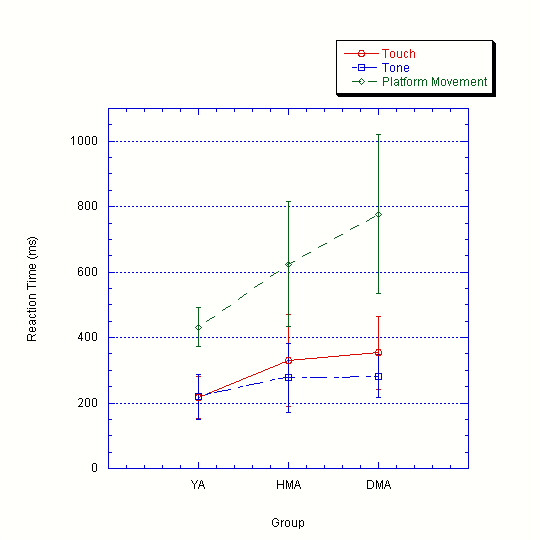
**Reaction Times to Plantar Touch, Auditory Tone, and Whole Body Lateral Perturbation by Group. **Average reaction times of each modality for each group are shown. YA = Young Adult; HMA = Healthy Mature Adult; DMA = Diabetic Mature Adult. Note reaction times for platform movement are significantly longer for each group and also increase from young healthy adults to healthy mature adults and diabetic mature adults.

Reaction times to platform movement (4 mm at 100 mm/s^2^), plantar touch, and a bell tone were measured in all subjects and can be seen in Table 1 and Figure 1 (See Attached File). Measurements for reaction times were taken as the time between the beginning of the stimulus and the button press indicating subjects detected the stimuli. Averages were taken from all trials that were detected.

For platform movements at 4 mm at 100 mm/s^2^, reaction times of all groups are significantly different (p < 0.05) from each other, with reaction times in the adults with diabetes being longest (mean = 777.8 ms), followed by aged matched adults (mean = 623.9 ms). Young adults had the shortest reaction times (mean = 431.0 ms) to movements.

For the touch modality, reaction times for adults with diabetes are significantly (p < 0.05) longer than both other groups (mean = 353.1 ms). However, reaction times to foot sole touch between young (mean = 216.0 ms) and healthy mature adults (mean = 331.5 ms) were not significantly different.

For the tone modality, no significant differences in reaction times were found between groups (diabetic mean = 282.6 ms; healthy adult mean = 276.9 ms; younger adult mean = 218.0 ms). For all groups, movement reaction times were significantly longer than the other two modalities (plantar touch and auditory tone), which did not differ significantly.

## Discussion

A reaction time measurement includes the latency in the sensory neural code traversing peripheral and central pathways; perceptive, cognitive and volitional processing; a motor signal again traversing both central and peripheral neuronal structures; and finally, the latency in end effector (e.g., muscle) activation. Unless there is a greatly lessened sensitivity or loss of the sensory receptors for a given modality, a stimulus well above perceptual threshold, will, by its very nature of being superthreshold, produce a strong neuronal signal in the peripheral nerve subserving the location stimulated. And, conversely once the volitional decision is made to signal that an event has been detected, the motor output emanating from the spinal cord to the finger that will press the signaling button should likewise be robust.

Since reaction time measurements have a central component, any decline, including those seen in normal aging, could indicate the presence of a peripheral and/or central neuropathy. Thus, it becomes difficult to tease out peripheral versus central effects when reaction times are slowed. But if the effect of a peripheral neuropathy is known through knowledge of the change in sensory and motor nerve conduction velocities brought about by the neuropathy, the extent to which the neuropathy will slow the reaction time can be estimated.

A lateral platform translational perturbation invokes many senses. These include, but are not limited to pressure and rapidly adapting tactile sensors in the feet and toes, proprioceptive sensors from the ankle and hips (assuming that lateral moves do not affect the knees), kinesthetic sensors in the muscles (muscle force from Golgi tendon organs, reflex activation form spindles, and an ill-defined sense of "perceived exertion", vestibular activation, and certainly visual stimuli (unless occluded via blindfold as we have done in this study), Because of the involvement of all of these "senses," attributing the cause(s) of a slowed platform perturbation reaction time to peripheral and central neuronal changes is difficult. But, if peripheral conduction velocities are known, as are reaction times to more "purer" sensory inputs such as foot sole touch or an auditory tone, then this latter knowledge can be factored into platform reaction time calculations to help tease out peripheral and central effects.

At this point, it is instructive to review the results reported in a preceding section of this paper and in Table 1, and then to call out the various findings to build a specific hypothesis. The key results follow:

1) Subjects with controlled type II diabetes all had mild, but measurable peripheral neuropathies in at least one nerve in the lower limb, while those without diabetes of age >50 had no measurable evidence of neuropathy;

2) Subjects with diabetes had increased reaction times to all three test modalities. Touch and Tone reaction times were slightly, but not significantly, higher, while platform reaction time was significantly higher.

3) Older adults, whether diabetic or not, had longer reaction times to platform moves and to foot sole touch (all locations) than did younger adults, and reaction times to the bell tone did differ between groups, even though those with diabetes had higher auditory air conduction thresholds at every frequency (except 8 kHz) tested than their non-diabetic counterparts.

4) Reaction times to platform movement are 200 to 300% longer in all groups when compared to reaction times to touch and tone;

### Implications

Individuals with diabetes often have neurological side effects that affect the peripheral nervous system. However, the increase in whole body movement reaction time seen in adults with diabetes in this study can not solely be related to peripheral nervous system changes due to diabetes. Even when motor nerve conductions slow from 50.0 m/s to 40.0 m/s (as seen in nerve conduction testing here), signal transmission time for a 1 m long nerve increases only 5 ms, which does not account for a 200 ms increase in movement reaction time. An additional slowing has to be occurring in the processing of the signals by the central nervous system.

Deficits in the central nervous system (CNS) of those with diabetes may also be seen in cognitive deficits. Dey, et al. found no correlation between the duration of diabetes and cognitive function in those with non-insulin-dependent diabetes less than 18 years old [31]. They hypothesized that in order to see the decline in cognitive function and other central nervous system effects seen by other researchers [10-13], a longer duration of disease state must be present. However, in our study, diabetics, all with less than 10 years disease duration had a significantly higher reaction time to movement, which could be interpreted to indicate that not only are central effects present, but they manifest themselves early in the disease. These increases in movement reaction times among the mature adults with diabetes may also have an effect on posture and gait. The longer reaction times of a slipping diabetic subject will thus increase the probability of a fall. Diabetics have been shown to have a higher incidence of postural instability [21-26], longer reaction times, and reduced peripheral sensations thus leading to a higher incidence of falls resulting from slips.

Reaction times to plantar surface touch indicate the extent of peripheral neuropathy in the population of diabetics. The fact that the mature adults with diabetes had increased reaction times to plantar touch is another indication that peripheral neuropathy was present in these subjects. However, we can see that the peripheral neuropathy of these adults with diabetes was not severe through the measurements of the Semmes-Weinstein monofilaments and sensory nerve conduction velocities. This increase may also play a role in the reaction time increase seen in the platform movement. If the subjects were unable to sense the movement for an additional 100 ms, then the 200 ms increase seen in adults with diabetes could be attributed to this sensory reaction time deficit, plus an increase in signal transmission through the nerves of approximately 5 ms, and an unknown cognitive slowing.

Auditory reaction times measured here for diabetics and age-matched controls do roughly concur with the one reaction time study that includes diabetics [5]. Although no significant differences in auditory reaction times were seen between mature adults with and without diabetes and their young adult counterparts, a sensorineural hearing loss was seen in the mature adults with diabetes at the mid- and high-frequencies. Controversy over the relationship between diabetes mellitus and sensorineural hearing loss has had a long history. Some authors have concluded that no correlation exists [32-35], yet others find significant correlations between diabetes and loss in the low [36], mid [36,37], and high [37] frequency ranges. This loss has been attributed to changes in the peripheral portion of the auditory pathway, because no change in signal conduction along the central auditory pathway in patients with diabetes has been seen [5]. Mean hearing thresholds tested here were consistent with those published in Tay, et al. [36] for healthy elder subjects. However, subjects with diabetes in this study had slightly higher thresholds than those published in Tay et al, but the higher thresholds were more consistent with those published by Celik, et al. [37]. No auditory evoked potentials were measured, therefore the source of the dysfunction (be it the central or peripheral auditory pathway) cannot be determined.

## Conclusion

From this study we can conclude that diabetes does affect reaction times, although the type and severity of the slowing may be related to the difficulty of the task and the prevalence of central and peripheral nerve deficits seen as side effects of diabetes. Auditory reaction times, the simplest of the tasks here with the shortest path between peripheral and central nervous system, did not show any differences in reaction times. When using a test that has a significantly longer path in the peripheral nervous system, such as the reaction time to plantar touch, slightly longer reaction times are seen in the adults with diabetes. When a more complicated task including detecting movement, signal transmission and interpretation, and response was required from the body, as in the platform movement reaction time test, a significant difference in reaction times were seen among all groups. This test takes more fully into account the peripheral nervous system signaling as well as the central nervous system processing and thus is a better overall test to determine deficits in healthy aging and aging individuals with diabetes.

We have presented here, in addition to normal auditory and touch reaction times, lateral whole body reaction time, which has been shown to be the most sensitive indicator of differences between healthy young, healthy mature adults, and mature adults with mild diabetes among the modalities tested here. In other studies, we have found that adults with diabetes have substantially higher thresholds than healthy adults to detecting whole body motion [29]. This, in addition to the increased whole body reaction times, indicate that mild diabetes has profound effects on ability to detect and react to motion, which leads to insights on their ability to detect and prevent slips and falls.

With the data presented here, it is impossible to determine the relative contribution of peripheral and central neural processes to the slowing seen on the whole body reaction time test. To determine this exact relation, authors are currently working on measures of cognitive processing that may provide more insight.

## Authors' contributions

SJR aided in the experimental design, carried out the data acquisition, data analysis and data interpretation, and drafted the manuscript. CJR aided in the experimental design, built the set-up, and aided in the final manuscript. JS completed the statistical data analysis and prepared all the tables and figures.
